# A Review of Calcineurin Biophysics with Implications for Cardiac Physiology

**DOI:** 10.3390/ijms222111565

**Published:** 2021-10-26

**Authors:** Ryan B. Williams, Christopher N. Johnson

**Affiliations:** 1 Department of Chemistry, Mississippi State University, Starkville, MS 39759, USA; rbw221@msstate.edu; 2 Center for Arrhythmia Research and Therapeutics, Vanderbilt University Medical Center, Nashville, TN 37232, USA

**Keywords:** calcineurin, phosphatase, protein phosphatase 2B (PP2B), calcium signaling, calmodulin, cardiomyocyte, cardiac physiology, hypertrophy

## Abstract

Calcineurin, also known as protein phosphatase 2B, is a heterodimeric serine threonine phosphatase involved in numerous signaling pathways. During the past 50 years, calcineurin has been the subject of extensive investigation. Many of its cellular and physiological functions have been described, and the underlying biophysical mechanisms are the subject of active investigation. With the abundance of techniques and experimental designs utilized to study calcineurin and its numerous substrates, it is difficult to reconcile the available information. There have been a plethora of reports describing the role of calcineurin in cardiac disease. However, a physiological role of calcineurin in healthy cardiomyocyte function requires clarification. Here, we review the seminal biophysical and structural details that are responsible for the molecular function and inhibition of calcineurin. We then focus on literature describing the roles of calcineurin in cardiomyocyte physiology and disease.

## 1. Identification and Nomenclature

**Figure 1:**
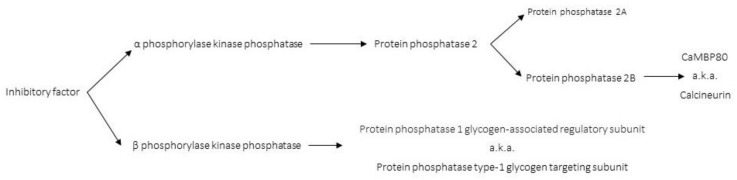
Diagram depicting the development of CaN nomenclature. Also known as (a.k.a.).

Calcineurin (CaN) was initially identified by two independent research laboratories [1,2]. In 1976, Wang et al. demonstrated that bovine brain cyclic nucleotide phosphodiesterase (likely PKA and/or PKG) required a higher concentration of a “calcium regulated protein modulator” (one of the original names for the calcium (Ca^2+^) sensing protein calmodulin) for activation in brain tissue compared to heart tissue. This was due to the presence of an “inhibitory factor” (CaN) in the brain preparations that counteracted the activation of the phosphodiesterase (Figure 1) [2]. Concurrently, in 1976, Antoniw et al. demonstrated that two distinct enzymes dephosphorylated the independent subunits of phosphorylase kinase [1] (phosphorylase kinase activates glycogen phosphorylase to release glucose-1-phosphate from glycogen [3]).

β phosphorylase kinase phosphatase is now known as protein phosphatase 1 glycogen-associated regulatory subunit, or protein phosphatase type-1 glycogen targeting subunit [4]. The α-phosphorylase kinase phosphatase (later discovered to be comprised of two similar enzymes, one of these being CaN, Figure 1) was shown to be an inhibitor of cyclic nucleotide phosphodiesterase [1,2], adenylate cyclase [5,6], turkey gizzard myosin light chain kinase [7], erythrocyte calcium magnesium ATPase [8,9], and phosphorylase b kinase [10]. While the mechanisms were not explicitly identified, inhibition likely occurs by CaN outcompeting and exhausting calmodulin availability and/or dephosphorylation of the enzyme substrate (α-phosphorylase kinase).

In 1979, the name calcineurin was coined by Klee et al. due to the “inhibitory factors” of calcium-binding properties and prevalence in the nervous system [7], though it took a few years before the name was widely adopted (Figure 1). In 1981, Helmreich et al. renamed alpha phosphorylase kinase phosphatase to Protein Phosphatase 2 (Figure 1) [11]. Shortly after, Stewart et al. resolved Protein Phosphatase 2 into two fractions (PP2A and PP2B). Both PP2A and PP2B (CaN) can dephosphorylate the α and β subunits of phosphorylase kinase; however, there are enzymatic differences between these two proteins. PP2A is promiscuous in substrate selection and dephosphorylates the phosphorylase kinase α subunit more rapidly (4–5 fold) than the β subunit. Conversely, PP2B is more specific in its substrate selection, but also dephosphorylates the phosphorylase kinase α subunit more rapidly (100 fold) compared to the β subunit [11].

## 2. Metal Binding and Calcineurin Activity

In 1982, Stewart et al. demonstrated that CaN (isolated from bovine neuronal tissue) has high affinity (K_d_ ≤ 10^−6^ M) for calcium in the presence of physiological concentrations of other divalent cations (i.e., Mg^2+^ and Mn^2+^). Moreover, 8–12% of maximal CaN activity can be obtained by calcium saturation (~pCa 5.8). An additional 10-fold increase in CaN activity was observed for the same calcium titration in the presence of calmodulin [12]. In 1984, two separate labs investigated the activity of CaN in the presence of other divalent cations. Li and Chan demonstrated that full activity of brain CaN requires Ca^2+^, calmodulin, and another divalent cation (Mg^2+^, Mn^2+^, Co^2+^, or Ni^2+^) [13]. Using a p-Nitrophenyl Phosphate (pNPP) assay, Pallen and Wang found that Ni^2+^ and Mn^2+^ can activate CaN in the absence of calmodulin, and addition of calmodulin further enhanced this CaN activity. Other divalent cations (Co^2+^ >> Ca^2+^ > Sr^2+^, Ba^2+^) require the presence of calmodulin to exert significant enhancement of CaN activity. Interestingly, Cu^2+^ is a better activator of CaN in the absence, rather than the presence, of calmodulin. Moreover, Be^2+^, Cd^2+^, Fe^2+^, Mg^2+^, Al^3+^, and Fe^3+^ do not stimulate CaN activity in the absence or presence of calmodulin. Lastly, Zn^2+^ inhibits the activation of CaN by other cations in both the absence and presence of calmodulin [14]. In 2004, Ping et al. investigated specific components of CaN in order to identify a mechanism for divalent cation activity enhancement. Their results revealed that the mechanism of divalent cation CaN activity enhancement is complex and likely involves several domains of CaN [15]. It is worth noting that an iron atom was found in the active site of the crystal structure determined by Kissinger et al. in 1995 (reviewed below). The iron is utilized for hydrolytic cleavage of phosphate from CaN substrates [16].

## 3. Kinetic Descriptions of the Calcium–Calmodulin–Calcineurin Interaction

A decade after identification, quantitative biophysical properties of CaN were reported. In 1987, Hubbard and Klee provided insight into the calmodulin-CaN complex lifetime by determining the association (k_on_ = 8.9 × 10^3^ M^−1^ s^−1^) and dissociation (k_off_ = 8.5 × 10^−5^ s^−1^) rates. Moreover, they calculated a dissociation constant for the calmodulin-CaN interaction (K_d_ ≤ 0.1 nM). The dissociation rate (k_off_) of calmodulin from the CaN complex was shown to be dependent on free calcium concentration ([Ca^2+^]_free_). A half maximal dissociation rate (k_off_) was achieved at 700 nM [Ca^2+^] [17]. This suggests that CaN has potential to function as a cardiac modification protein on a beat-to-beat basis, as this [Ca^2+^] would provide a maximal calcium-sensing ability in between diastolic (0.1 μM) and systolic (1.0 μM) [Ca^2+^] (K_d_ defines the inflection point which has the steepest slope, i.e., the most sensitive response within the sigmoidal calcium binding curve). In 2005, Quintana et al. utilized stopped-flow fluorescence spectroscopy to determine the association and dissociation rates of the full length CaN-calmodulin interaction in the presence of calcium (4.6 × 10^7^ M^−1^ s^−1^, 1.2 × 10^−3^ s^−1^, and calculated K_d_ of 28 pM). Moreover, a dissociation rate for the isolated CaN A subunit-calmodulin interaction in the presence of calcium was determined (2.6 × 10^−4^ s^−1^) [18]. In 2009, Kilka et al. reported CaN dephosphorylation activity (K_m_ and k_cat_) for NFAT, DARP-32, Elk-1, Tau, and RII peptide by α, β, and γ CaN using ^33^P-labeled substrates [19]. These results indicate that substrate processing is likely the rate limiting step of the CaN dephosphorylation process, as the rapid calcium–calmodulin kinetics occur on a much more rapid timescale.

## 4. Isoforms of Calcineurin

There are three isoforms of the CaN A subunit (CaN A) (α, β, and γ) [20,21] and two isoforms of the B subunit (CaN BI and CaN BII) [22]. In the heart, the CaN A subunit isoforms (α and β) can interact with CaN BI [22]. The CaN Aγ isoform is predominantly found in the testis and can interact with CaN BII [20,23,24]. We speculate that early CaN studies primarily characterized the α isoform of CaN A due to its abundance in neuronal tissue, which was the primary source of CaN before recombinant protein expression.

## 5. Autoinhibition of Calcineurin Activity

**Figure 2:**
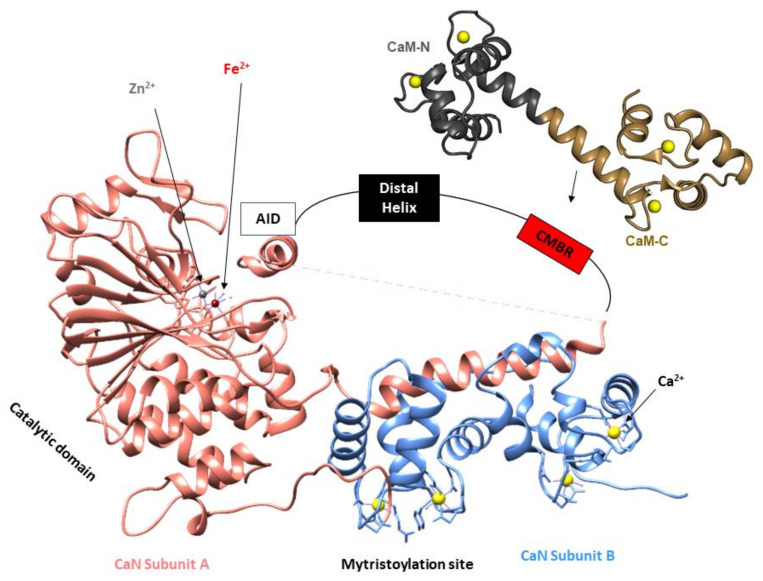
Diagram illustrating the key components of the CaN heterodimer (developed from PDB ID: 1AUI [16]). CaN A shown in salmon, CaN B shown in blue, Auto-Inhibitory Domain (AID), Calmodulin-Binding Region (CMBR). Due to the disordered nature of the regulatory domain, this region has been represented by a line and boxes for the distal helix and CMBR. Zn and Fe are located at the active site for dephosphorylation of CaN substrates. The myristoylation site located at the N-terminal region of the CaN B subunit is cleaved so the two CaN subunits can form a heterodimer [28]. Ca^2+^ binding to the CaN B subunit can evoke a small enhancement of enzymatic activity in the absence of CaM. Ca^2+^–CaM–CaN interaction enriches CMBR α-helical content resulting in translocation of the autoinhibitory domain which leads to enhancement of CaN activity [29].

In 1979, Klee et al. demonstrated that CaN was comprised of two polypeptide chains (CaN A, MW = 61,000 Da and CaN B, MW = 15,000 Da), and these two subunits form a 1:1 heterodimer (Figure 2). The A subunit interacts with calmodulin in a calcium-dependent manner, and the B subunit directly binds calcium [7]. In 1983, Manalan and Klee went on to demonstrate that, similar to other calmodulin-stimulated enzymes, CaN can be activated and rendered calcium–calmodulin-independent by limited proteolysis. Moreover, they found calmodulin can protect CaN from degradation in the presence of Ca^2+^. Based on their tryptic digest data, Manalan and Klee concluded that CaN contained a long intrinsically disordered region connected to a small-structured domain (Auto Inhibitory Domain (AID)) that was required for blunting enzyme activity. This was the first report of an autoinhibitory mechanism that described CaN auto-inhibition [25]. Shortly after, Aitken et al. posited the CaN B subunit shares structural similarity with calmodulin and troponin C, as it has four Ca^2+^ binding EF-hand motifs (based on sequence analysis and Garnier’s method of secondary structure prediction) [26,27].

## 6. Enhancement of Calcineurin Activity

CaN activity can be modified by several mechanisms. In 1994, Stemmer and Klee demonstrated that CaN B and calmodulin activate the phosphatase by different yet complementary processes [29]. In the absence of calmodulin, Ca^2+^ can stimulate a small but significant level of CaN activity. At < 10 nM [Ca^2+^], isolated CaN is inactive, and at ~500 nM [Ca^2+^], 10% of maximal activation is achieved. Calmodulin binding in the presence of Ca^2+^ further stimulates CaN an additional 10-fold. Intriguingly, Ca^2+^ binding to CaN B is a prerequisite for calmodulin enhancement. Stemmer et al. also posited that CaN B and calmodulin play two different roles in calcium stimulation. Ca^2+^ binding to calmodulin results in the disruption of the autoinhibitory domain from the active site and an increase in V_max_. Ca^2+^ binding to CaN B increases the affinity of CaN for its substrate [29].

Notably, the stoichiometry of active CaN was the subject of some controversy. In 2006, Ye et al. reported a crystal structure displaying a 2:2 stoichiometry between calmodulin and a peptide corresponding to the calmodulin binding domain of CaN. The protein construct used for this crystallization was comprised of only 25 amino acids of CaN (corresponding to the calmodulin binding site [30]) covalently attached to calmodulin by a 5-glycine linker [31]. In 2008, Ye et al. repeated this investigation without the 5-glycine linker and reported a nearly identical 2:2 stoichiometric structure [32]. In 2014, Dunlap et al. also reported an X-ray crystallography structure of the calmodulin–CaN interaction. The stoichiometry of calmodulin binding to this construct (regulatory domain of the catalytic subunit of CaN (residues 373–468) revealed a 1:1 stoichiometry which was also confirmed by time-resolved fluorescence and size exclusion chromatography [33].

The mechanistic details for how calmodulin enhances CaN activity have been the subject of much investigation. In 2012, Rumi-Masante et al. leveraged hydrogen–deuterium exchange rates to demonstrate that the autoinhibitory domain of CaN is connected to the main catalytic subunit by an intrinsically disordered region. Based on calmodulin’s interaction with this region, the region has been referred to as the “regulatory domain” (Figure 2). Ca^2+^–calmodulin binding to this regulatory domain enriches the alpha helical content leading to subsequent enzyme activation [34]. In 2013, Dunlap et al. confirmed these findings and further demonstrated that the Ca^2+^–calmodulin–CaN interactions reduce the distance between the two termini of the regulatory domain [35].

In 2016, Cook and Creamer demonstrated that molecular crowding increases the melting temperature of the α-helical content of the CaN distal helix. Correspondingly, they observed that increasing molecular crowding, by adding dextran 70 or ficol 70, enhanced enzyme activity [36]. Increasing the α-helical content of the regulatory domain facilitates dislocation of the autoinhibitory domain. In 2017, Yadav et al. reported solution NMR resonance assignments of the regulatory domain of CaN. These chemical shift values were consistent with the CaN regulatory domain containing a high percentage of disordered content. In-depth analysis revealed that a small amount of secondary-structure helical propensity may be present in the CaN regulatory domain in the absence of calcium–calmodulin [37]. In 2018, Sun et al. leveraged molecular dynamics simulations to propose a mechanism where residues outside of the calmodulin-binding region contribute to enzyme activation [38]. The complete mechanistic details governing Ca^2+^-calmodulin enhancement of CaN are still the subject of active inquiry [38,39].

Intriguingly, several aspects of calmodulin modification of CaN appear to parallel other systems. The release of an autoinhibitory domain upon calmodulin binding is similar to the activation mechanism of calmodulin-dependent protein Kinase II (CaMKII). In this mechanism, Ca^2+^-activated calmodulin binds to CaMKII which releases domains from the central hub region and allows for enzymatic activity [40]. Moreover, this type of mechanism (release of autoinhibition) may also apply to ion channels. In 2018, Johnson et al. demonstrated that calmodulin binding to the inactivation gate of the cardiac sodium channel promotes recovery from an inactivated state which would allow for enhancement of sodium channel activity [41].

## 7. External Inhibition of Calcineurin Activity

Cyclosporin A (CsA) is and has been commonly used to prevent organ transplant rejection (kidney, heart, bone marrow, and liver) in humans since the 1980s. Prior to the early 1990s, the molecular mechanism was largely unknown and therefore has been the subject of much investigation. Many scientists also use CsA and Tacrolimus (also known as FK506) to inhibit CaN activity in vivo. In 1989, Kay et al. demonstrated that lymphocyte activation (induced by Ca^2+^ ionophores) can be suppressed by 0.1 nM FK506 [42]. Concurrently, Tropschug et al. observed that cyclophilin is required for the CsA inhibitory effect. Moreover, they posited that binding of CsA to cyclophilin leads to the formation of a complex that interacts with an “unidentified cellular component” (later shown to be CaN) [43]. Shortly after, Randak et al. found that CsA-treated cells lack an interaction between “lymphocyte-specific factors” (later shown to be CaN and NFAT, see Section 10) and the interleukin-2 enhancer [44].

In 1991, Lin et al. demonstrated that FK506 exerts an inhibitory effect during early events of T-cell activation in a manner indistinguishable from CsA. Moreover, the pathway that was inhibited by FK506 and CsA required a measurable rise in intracellular [Ca^2+^] [45]. At the same time, Friedman et al. and Liu et al. found that the CsA-cyclophilin and FK506-FKBP12 complexes directly bind to and inhibit CaN in vitro [46,47]. Fruman et al. built upon these findings and demonstrated that CsA and Tacrolimus lead to abolished CaN activity within Thymus (T)-cells [48]. These seminal studies were the first to report a common mechanism of CaN inhibition. We note that the cyclophilin and FKBP12 proteins have other roles in cellular physiology [49], and these require further investigation (for a description of how CaN inhibition leads to a decreased immune response, see Section 7.2).

### 7.1. Other Mechanisms of Calcineurin-NFAT Inhibition

CaN activity can be impaired by several mechanisms (Table 1), such as modification of the active site metal properties or the substrate-binding interface. In 2001, Namgaladze et al. demonstrated that superoxide can inhibit CaN activity by reducing Fe^3+^ at the active site. Moreover, they observed that nitric oxide can block superoxide’s ability to inhibit CaN activity [50]. In 2004, Roehrl et al. identified several small organic molecules that inhibit CaN’s ability to activate the Nuclear Factor of Activated T-cells (NFAT). Their findings were unique as this was a direct alteration to the CaN-NFAT interaction [51], whereas CsA-cyclophilin and FK506-FKBP12 achieved inhibition by blocking the CaN-active site [46,47]. Roehrl’s innovative strategy is notable as these small molecules achieve inhibition of the CaN-NFAT pathways without altering other known cellular functions of CaN [51].

In 2005, Kang et al. went on to demonstrate that several small molecules can allosterically achieve inhibition of CaN-NFAT association through interactions outside of the consensus NFAT binding site (see Section 9) [52]. In 2014, Qian et al. produced a high affinity (K_d_ = 2.6 nM) inhibitor of the CaN-NFAT interaction and showed that this peptide prevented NFAT translocation to the nucleus using confocal microscopy [53].

### 7.2. Structural Basis of Calcineurin Inhibition and Activity

Shortly after the molecular mechanism of CsA/FK506 was identified, the structural underpinnings of inhibition were described. In 1995, Griffith et al. reported a high-resolution X-ray crystal structure (2.5 Å) of neuronal bovine CaN bound to FKBP12-FK506 complex. To obtain well-diffracting crystals, samples were subjected to proteolysis for 3–4 days to remove disordered regions of the protein complex. This resolution provided well-resolved electron densities for the amino acid side chains at the FK506/FKBP12-CaN binding interface. Notably, this FKBP12-FK506 complex did not contact the phosphatase active site on CaN A. They conclude that inhibition occurs by the FKBP12-FK506 sterically occluding the substrate approach to the active site [62]. Several weeks later, Kissinger et al. reported a different structure of the CaN-FKBP12/FK506 construct. In this structure, the FKBP12-FK506 inhibitor forms an additional contact with CaN near the catalytic site. They posit that this additional interaction could allosterically induce subtle changes to the active site geometry, giving rise to inhibition [16].

**Figure 3:**
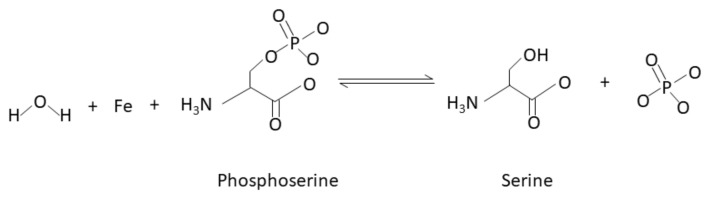
Chemical equation of dephosphorylation of a serine residue by an iron atom located in the CaN-active site adapted from Kissinger et al. (1995). A water molecule is deprotonated by the iron atom, followed by a hydrolysis reaction between the deprotonated (activated) water molecule and phosphorylated serine residue (phosphoserine). This reaction yields serine and phosphate. Threonine substrates follow a similar mechanism of water activation and dephosphorylation [16].

Notably, these structural investigations also provide a rationale for substrate processing. In 1995, Kissinger et al. determined multiple high-resolution X-ray crystal structures; one of full-length human CaN (2.1 Å) and a second of full-length human CaN bound to FKBP12-FK506 (3.5 Å). SDS-PAGE electrophoresis indicated that both protein crystals contained intact CaN without proteolytic degradation. In the absence of the FKBP12-FK506 complex, the CaN autoinhibitory domain sterically occluded the Zn/Fe active site. This well-resolved isolated CaN structure indicated that dephosphorylation could occur by a catalytic nucleophilic attack on the substrate’s phosphate by a metal-activated water molecule (Figure 3) [16].

## 8. Endogenous Regulators of Calcineurin Activity

In 1995, Stemmer et al. found that crude rat brain extracts contain a CaN inhibitor that is likely of protein origin [63]. Subsequently, an endogenous protein inhibitor of CaN was identified. In 1998, two independent laboratories reported an endogenous inhibitor of CaN and each reported a different name: calcineurin inhibitor (cain) and calcineurin binding protein 1 (Cabin 1). Sun et al. demonstrated that the CaN-cain/Cabin 1 interaction is dependent on protein kinase C (PKC) and Ca^2+^ concentration. They observed that in the presence of a Ca^2+^ ionophore (ionomycin) and a PKC activator (PMA), CaN-dependent luciferase activity is nearly abolished [57]. Concurrently, Lai et al. demonstrated that cain/Cabin 1 noncompetitively inhibits CaN [56].

Similar to the exogenous inhibition, endogenous inhibition can occur by multiple mechanisms. In 2009, Mehta et al. observed that the human protein RCAN (regulator of CaN) can either stimulate or inhibit CaN activity. RCAN stimulation of CaN required Glycogen Synthase Kinase 3 (GSK-3) and the E3 ubiquitin ligase SCF^Cdc4^. RCAN inhibition required a conserved CaN docking site (PxIxIT-like amino acid motif, see Section 9). There are three types of RCAN proteins (RCAN1, RCAN2, and RCAN3) [64]. In 2009, Mulero et al. developed an in vitro high-throughput fluorescence polarization assay that utilizes an RCAN1 peptide for identifying molecules that have immunosuppressant potential [65]. In 2015, Kim et al. demonstrated that protein kinase A indirectly inhibits CaN activity through RCAN1 using an NFAT luciferase assay [66]. Recently, Li et al. utilized a combination of structural, biophysical, and biochemical techniques to describe RCAN1 inhibition of CaN activity. Specifically, they found that RCAN1-CaN inhibition occurs by multiple mechanisms: block of substrate recruitment sites and block of the CaN active site [67].

AKAP79 is often used as a complementary control to CsA and FK506 for CaN inhibition. In 2012, Li et al. observed that the anchoring site of AKAP79 (a scaffolding protein mainly found in neurons) interacts with the same surface of CaN as the PxIxIT motif of NFAT. Moreover, they found that AKAP79 can promote or hinder NFAT activation depending on the CaN AKAP79 affinity [68].

## 9. Calcineurin Substrate Binding

In 1982, Aitken et al. determined that the N-terminus of the CaN B subunit contains a myristoyl group [28]. It is now known that myristoylation is widely utilized by many proteins for membrane targeting [69]. It remains to be determined if and how myristoylation influences or alters CaN’s localization and/or substrate specificity. We note that localization of CaN to specific cellular compartments could influence substrate selection. In 1994, Donella-Deana et al. demonstrated (using small 6–32 amino acid peptides) that the sequence surrounding the substrate’s phosphate does not always provide a clear signature for CaN-substrate activity [70].

### 9.1. Calcineurin-Binding Region 1 (CNBR1) and the PxIxIT Motif

Over the past several decades, there has been a growing interest in CaN substrate recognition. Thus far, two binding motifs have been identified. In 2004, Li et al. demonstrated that several CaN targets (such as NFAT) utilize a common amino acid sequence for interaction (PxIxIT motif (also termed CaN Binding Region 1 [CNBR1] in 1998) [71]) [72]. In 2007, Roy et al. found that CaN binds with varying weak affinities to small peptides containing variations of the PxIxIT motif. CaN, like other signaling enzymes, does not typically form stable complexes with its substrate. Intriguingly, weak CaN substrate-affinity conflicts with achieving selective protein post-translational modification [73].

In 2007, Li et al. provided a structural characterization that yielded insight into the molecular requirements of CaN PxIxIT motif interactions. An X-ray crystal structure of CaN bound to a 14 amino acid peptide containing the PVIVIT sequence provided an atomic resolution description of a CaN-substrate interaction and revealed the important side chains used for binding. Alterations to these substrate amino acids significantly impaired CaN binding in vitro which confirmed the observed crystal contacts. Notably, the PVIVIT sequence generated the strongest of the series of PxIxIT interaction affinities and substrate affinity influenced CaN’s response to [Ca^2+^] [74]. This is reminiscent of Ca^2+^-calmodulin binding affinity being tuned by substrate interaction [75].

### 9.2. Calcineurin Binding Region 2 (CNBR2) and the LxVP Motif

In addition to the PxIxIT motif, CaN utilizes a second binding motif that differs in key binding properties from the PxIxIT motif. In 1999, Liu et al. identified a second binding region for CaN-NFAT interaction (CaN Binding Region 2 (CNBR2)) using a GST-pulldown assay with Western blot analysis [76]. Concurrently, Park et al. also reported a second binding site for the CaN-NFAT interaction using a Secreted Alkaline Phosphatase (SEAP) assay [77]. Soon after, Liu et al. found that a shorter 16 residue peptide (Pep3 derived from CNBR2) was responsible for the CNBR2 interaction [78]. In 2006, Martı’nez-Martı’nez et al. provided a detailed comparative analysis of CaN binding activity between PxIxIT and CNBR2 (now known as the LxVP docking site). Consistent with data from Liu et al. (1999), Park et al. (2000), and Liu et al. (2001), Martı´nez-Martı´nez et al. demonstrated that the NFATc1 LxVP motif binds CaN more efficiently than PxIxIT motifs [79]. We note that these investigations utilized NFAT binding sites. Further investigation is required to delineate if other CaN substrates have PxIxIT and/or LxVP binding preference.

## 10. An Overview of Calcineurin Function in Cardiac Physiology

The most prominent and well-recognized CaN signaling pathway is attributed to the activation of nuclear factor of activated T cells (NFAT). While not the focus of this review, a brief description of the CaN-NFAT interaction has been provided to understand many of the investigations that are focused on the roles of CaN in cellular functions. Dephosphorylation of cytosolic NFAT (NFAT_c_) facilitates translocation of the CaN-NFAT_c_ complex to the nucleus, which promotes synthesis of nuclear NFAT (NFAT_N_) protein [80]. An interaction between NFAT_C_ and NFAT_N_ proteins lead to production of interleukin-2 (IL-2) (cytokine that is used for an immune response). For a focused review of CaN’s role in NFAT signaling, see Hogan 2017 [81].

### 10.1. Calcineurin Is Essential for Cardiac Development, Cardiomyocyte Stress Response, and Cardiac Physiology

CaN has a prominent role in cardiac development. In 2010, Maillet et al. demonstrated that cardiac-specific deletion of CaN was lethal for mice one day after birth. Specifically, they found defects in right ventricular development, reduced ventricular trabeculation septal defects, and valvular overgrowth [82]. CaN also appears to have a role in adult cardiac physiology. CaN deletion (alpha myosin heavy chain Cre-expression dependence) reduced cardiac contractility, increased incidence of arrhythmia, and reduced cardiac myocyte content [82]. Moreover, in 2006, Bukowska et al. demonstrated that the CaN A subunit (β isoform) was significantly upregulated in human atrial tissue from patients with sinus rhythm and chronic persistent atrial fibrillation [83].

CaN has also been shown to have a variety of other roles in cardiomyocytes as well as other cell types. Moreover, several lines of evidence suggest that hypertrophy impairs physiological calcineurin-ion channel regulation (discussed in Section 11). For a comprehensive overview of direct interactions between CaN and cardiac proteins, see Table 2. Interestingly, CaN is also an effector for several proapoptotic kinases such as Apoptosis Signaling Kinase 1 (ASK1) and c-Jun N-terminal protein kinase (JNK1/2) [84,85,86]. These kinases are important for physiological viability of cells. ASK1 is an upstream promotor of apoptosis that activates in response to proapoptotic stimuli. Upon dephosphorylation of ASK1 by CaN, ASK1 disassociates with 14-3-3 protein [87]. Downstream to this dephosphorylation event, a variety of other kinases are activated such as JNK1/2 [88]. At the end of this signaling cascade, apoptosis is induced. For a review of ASK1 signaling with insights on prolonged JNK activation, see Ogier et al. (2020) [89].

### 10.2. Calcineurin, Cardiac Ion Channels, and Ion Pumps

Given data presented by Bukowska et al. [83] and Maillet et al. [82], we have surveyed the literature to understand the potential of CaN regulating or modifying cardiac ion channels and pumps. While many of the investigations have explored CaN-ion channel modification relationships for systems outside of a cardiomyocyte, there are several lines of evidence that suggest CaN-ion channel regulation contributes to cardiac physiology.

In 2011, Prasad and Inesi demonstrated that CaN A can alter production of SERCA 2 leading to changes in cytosolic [Ca^2+^]. Neonatal rat ventricular cardiomyocytes with silenced genes for either the α or β isoform of CaN A displayed reduced SERCA 2 expression. Application of thapsigargin (SERCA 2 inhibitor) rescued SERCA 2 expression (mRNA and protein) and restored Ca^2+^ transport. Importantly, these effects were reproduced using ionomycin in place of thapsigargin. This is consistent with a mechanism where an increase in diastolic Ca^2+^ activates CaN and restores SERCA 2 production. Additionally, there is evidence that CaN influences production of other important cardiomyocyte proteins. Cardiomyocytes exposed to CsA displayed lower mRNA content for phospholamban and Na^+^/Ca^2+^ exchanger [101].

CaN has been shown to modify the function of the cardiac L-type calcium channel in excitable cells. This channel has a pivotal role in establishing the cytosolic [Ca^2+^] through the calcium-induced-calcium-release mechanism. Changes in cytosolic [Ca^2+^] activate or inactivate many calcium-sensitive proteins and enzymes within a cell. In 1997, Schuhmann et al. observed that CaN alters the function of the L-type calcium channel in human umbilical vein smooth muscle cells [102]. Others have shown that CaN activity and ion channel function are correlated in diseased cardiomyocytes. In 2000, Saito et al. demonstrated that CaN and the L-type calcium channel play a critical role in isoproterenol-induced apoptosis of rat cardiomyocytes [96]. We note that further investigation of the physiological CaN-L-type calcium channel relationship is required as the molecular and cellular functions are likely different in a diseased or non-stressed state [103]. Much of the work surrounding CaN-L-type calcium channel modification relies on the CaN inhibitor CsA, and conflicting results are obtained from several laboratories. For further descriptions of these disparate results, we refer the reader to Wang et al., 2014 [103].

The heart the Na^+^/K^+^ ATPase may also be modified by CaN. In renal tubule cells, Aperia et al. demonstrated that stimulation of α-adrenergic receptors increased the activity of the Na^+^/K^+^ ATPase. Addition of FK506, a CaN inhibitor, was shown to blunt the effects of α-adrenergic receptor stimulation to the Na^+^/K^+^ ATPase [90]. We note that the alpha1 beta1 isoforms contained in proximal convoluted tubules appear to be present in cardiomyocytes [104]. This raises the possibility that CaN may have a role in stimulating Na^+^/K^+^ ATPase activity in a cardiomyocyte.

An interaction between CaN and the ryanodine receptor (RyR2) has also been posited for modulating RyR2 Ca^2+^ release. In 2000, Bandyopadhyay et al. demonstrated CaN coimmunoprecipitates with RyR2 in a calcium-dependent manner [94]. We note that the RyR2 sequence contains 5 LxVP motifs which suggests CaN may contribute to RyR2 regulation. Moreover, CaN inhibitors (CsA and FK506) can modify RyR2 function, as Bandyopadhyay et al. report they can reduce the frequency of spark calcium release [94]. This data is further supported by Savoia et al. as they demonstrated in 2014 that application of a CaN autoinhibitory peptide reduces RyR2 calcium release frequency in airway smooth muscle cells [105].

### 10.3. Calcineurin Can Impart Different Effects to Ion Channel Function

It has been demonstrated that CaN is involved in the restoration of the resting membrane potential by dephosphorylating the Na^+^/K^+^ ATPase, resulting in Na^+^ retention [90,106]. Conversely, dephosphorylation of L-type calcium channel by CaN reduces ion channel conduction. We note the mechanistic details of this process are largely unknown [96,102]. In the case of the RyR2, it has been suggested that CaN anchors to the FKBP12 accessory protein associated to the receptor and then inactivates RyR2 by dephosphorylation. To the best of our knowledge, there is one report of this mechanism, and further investigation is required [94].

## 11. Calcineurin’s Roles and Contribution to Cardiac Disease

An extensive number of investigations have demonstrated that improper CaN activity can induce and/or contribute to heart disease. Inhibition or reduction of CaN activity is becoming an attractive approach for intervention [107,108]. Table 3 provides a comprehensive summary of CaN’s roles in cardiac pathophysiology. In addition to seminal findings, below we highlight some of the less appreciated mechanisms of CaN signaling. Lastly, we discuss CaN activity with consideration for hormone signaling and ion channel dysfunction.

### 11.1. Inhibition of Calcineurin Reduces Cardiac Hypertrophy

One of the most prominent pathological outcomes related to CaN is cardiac hypertrophy (thickening of the heart muscle). In 1998, Molkentin et al. demonstrated that addition of phenylephrine or angiotensin II with constitutively active CaN or NFAT increased brain natriuretic peptide (BNP) promoter activity (located in the heart). This increased BNP activity was shown to be dependent on the presence of CaN, NFAT, and GATA4 (a transcription factor involved in proper cardiac muscle development). This led to heart dilation and hypertrophy. Inhibition of CaN (CsA or FK506) prevented these effects [109]. The role of CaN in cardiac hypertrophy has also been investigated using other CaN inhibition strategies. In 2001, De Windt et al. demonstrated that genetic inhibition of CaN by cain/cabin1 or the AKAP79 inhibitory domain reduces cardiac hypertrophy in mouse models [113]. In addition to preventing hypertrophy, reducing CaN activity may also be advantageous for treating hypertrophic disease. In 2000, Taigen et al. demonstrated that CaN activity is increased upon the addition of hypertrophic stimuli (angiotensin II and phenylephrine) and application of CsA or FK506 rescued cardiomyocytes from hypertrophy [110]. This was unique as previous investigations from Molkentin et al. had explored the effects of overactive CaN without consideration for variable CaN activity [109].

While NFAT is the predominant mechanism identified for generating hypertrophy, there are many proteins that can trigger this process. Moreover, CaN modification can have different functional consequences in physiological and diseased states. In 2002, Sah et al. demonstrated that overexpression of the K_V_4.2 N-terminus increased CaN activity and heart to body weight ratio (changes in heart to body weight ratio are often attributed to cardiac dilation and/or hypertrophy). Addition of Verapamil (L-type calcium channel inhibitor that reduces cytosolic Ca^2+^ concentration [123]) reduced the heart to body weight ratio. CaN’s contribution to the phenotype was verified by application of CsA which removed the effects. Neither CsA nor verapamil altered heart to body weight ratio in wild-type mice, suggesting that physiological heart to body weight ratio is not reliant on CaN activity [116].

### 11.2. Constitutively Active CaN or NFAT Can Adversely Affect Ion Channels and Cardiac Function

In 2001, Yatani et al. demonstrated that models of constitutively active CaN exhibit enhanced L-Type calcium channel conduction. This led to cardiac hypertrophy and heart failure [114]. The mechanisms for this process remain to be elucidated, as CaN inhibitors such as CsA have yielded disparate results [103]. Under disease conditions, there is also evidence that CaN can modify potassium channels. In 2006, Gong et al. demonstrated that excessive CaN activity (constitutively active CaN) increases K_V_4.2 channel density through interactions with the K_V_4.2 gene. Addition of cain/Cabin 1 removed this effect [121]. Interestingly, contrary effects have been observed in other investigations, where hypertrophy impaired CaN-K_V_4.2 regulation. Specifically, Zobel et al. observed that stimulation of CaN (phenylephrine) resulted in a decreased K_V_4.2 current density [124]. This was subsequently supported by Panama et al. who demonstrated that CaN inhibition (via cain) can reduce mRNA of KChIP2 and Kv4.2 and the combination of phenylephrine/propranolol exacerbated this effect [125]. Further investigation is required to understand how excessive CaN activity imparts different effects to K_V_4.2 channel density.

CaN also modifies the production of other cardiac ion channel proteins. In 2006, Kuwahara et al. demonstrated that activation of CaN in a pressure-overloaded system can upregulate transient receptor potential proteins (TRPC6) in mouse and failing human hearts [126]. It has also been reported that CaN can form a complex with IP_3_R or RyR receptors through interactions with FKBP12. Moreover, inhibition of CaN with CsA alters the Ca^2+^ flux produced by ligand (IP_3_) activation of the ion channel [127].

In 2002, Petrashevskaya et al. demonstrated that constitutively active CaN or NFAT can create several adverse physiological effects. Critical modifications such as increased action potential duration, decreased heart rate, and decreased β-adrenergic stimulation have been observed [117]. Moreover, De Windt et al. demonstrated that genetic inhibition of CaN (overexpression of cain/Cabin 1 or the AKAP79 inhibitory domain) reduced isoproterenol induced hypertrophy [113]. Lastly, excessive inhibition of CaN during transaortic constriction leads to modification of the function of several key proteins involved in cardiac calcium cycling (phospholamban, RyR2, SERCA2a, and NCX) and promotes an increase in left ventricular end diastolic pressure and myocardial stiffness [122]. Given the abundance of ion channel targets that are modified by CaMKII, we note that under disease conditions (hypertrophic and constitutively active CaMKII), it has been demonstrated that CaMKII can phosphorylate CaN (S197) leading to reduced CaN activity [59]. Further work is required to understand the relationships between CaMKII and CaN function under physiological conditions.

### 11.3. CaN May Also Protect Cardiomyocytes from DNA-Damage-Induced Apoptosis

CaN activity stimulated by phenylephrine protected cardiomyocytes from staurosporine and 2-deoxyglucose induced cell death [112]. This appears to conflict with other reports that have implicated CaN in facilitating apoptosis [84,85,86]. We note that these investigations interrogate CaN using different adrenergic pathways (phenylephrine = α-adrenergic stimulation, isoproterenol = β-adrenergic stimulation). We speculate that similar to health and diseased states, the different adrenergic pathways may utilize CaN activity uniquely. For a depiction of the α-adrenergic stimulation pathway that results in pathophysiological hypertrophy, we refer the reader to Cotecchia et al., 2015 (Figure 1) [128].

### 11.4. Hormonal Connections to Calcineurin

Hormone signaling is an important modifier of cardiac physiology. Of the several hormones involved in regulating cellular homeostasis [129], estrogen has been demonstrated to modify CaN signaling in pathological T cells, neuronal cells, and heart cells. Mechanisms include estrogen receptor stimulation, alteration of CaN mRNA levels, and alterations to CaN compartmentalization or localization. For a more in-depth review of hormonal regulation of the heart, see Gordan et al., 2015 [130].

With recent reports identifying estrogen modification of CaN in cardiomyocytes [131,132], we briefly summarize estrogen-CaN findings in T-cells as this may shed light on estrogen-CaN cardiomyocyte signaling. In 1998, Rider et al. revealed that estrogen can alter the levels of CaN mRNA in human female lupus T cells. The effects were found to be dose- and time-dependent [133]. In 2000, Rider et al. also demonstrated that the estrogen receptor can impart gender-specific effects. Blocking female estrogen receptors (ICI 182,780) inhibited an increase in CaN mRNA and CaN activity in female patients. Moreover, this effect was not observed in male patient T cells [134], indicating that CaN has gender-specific physiological and diseased contributions. In 2008, Don Yi and Simpkins observed that in primary cortical neurons, glutamate (glutamate-induced apoptosis) decreases CaN and PP2A activity. Moreover, 17β-estradiol dampened glutamate’s ability to alter CaN activity [135].

In 2008, Pedram et al. used a cardiac fibrosis model (angiotensin II-induced) to demonstrate that 17β-estradiol indirectly inhibits CaN and prevents angiotensin II-induced fibrosis. Specifically, knockout of the estrogen receptor (β isoform) impaired 17β-estradiol’s ability to reduce angiotensin II enhancement of CaN activity. This data confirmed that the estrogen receptor β isoform is required for estrogen reduction of CaN activity as well as prevention or rescue of cardiac hypertrophy and fibrosis. Moreover, RCAN1 protein expression was found to be stimulated by 17β-estradiol [136], hinting at a possible mechanism for estrogen-mediated CaN inhibition in a cardiomyocyte. In 2009, Donaldson et al. demonstrated that estrogen hinders the rise in CaN A subunit expression that is induced by transaortic constriction or phenylephrine. Based on this data, they posit that 17β-estradiol and estrogen receptors reduce pressure overload cardiac hypertrophy by increasing CaN A degradation [137]. Further work is required to delineate the molecular and cellular mechanisms that underly hormonal CaN regulation in cardiomyocytes.

## 12. There Is Still Much Work to Be Done

While cellular and physiological descriptions of CaN function have been well described, there are several knowledge gaps that limit our understanding of CaN’s role in cardiac physiology and disease (Table 4). It will be of significant interest to characterize biophysical details of CaN complexes. Quantitative descriptions such as kinetic on and off rates, binding energies, and thermal stability of the CaN-substrate interactions will provide further insight into CaN-mediated signaling pathways. Elucidation of these properties will illuminate potential therapeutic targets and novel strategies for small molecule modification and intervention.

## Figures and Tables

**Table 1:** Calcineurin inhibitors. Exogenous contributors to CaN inhibition shown in red, endogenous proteins that contribute to CaN inhibition shown in blue.

Inhibitor	Structure	Molecular Effect	Usage	Ref.
Cyclosporin A-Cyclophilin A complex (CsA-CyA)	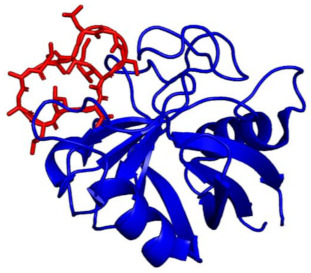 PDB ID: 1CWA; Red = Cyclosporin A, Blue = Cyclophilin A	CsA binding to cyclophilin A → Binding/inhibition of CaN	Clinical and experimental	[47,54]
Tacrolimus-FKBP12 complex (FK506-FKBP)	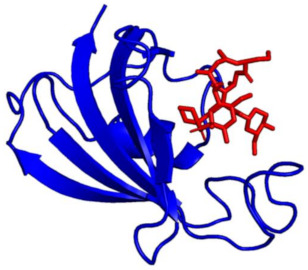 PDB ID: 1FKF; Red = FK506; Blue = FKBP12	FK506 binding to FKBP → Binding/inhibition of CaN	Clinical and experimental	[45,55]
Calcineurin inhibitor (cain) protein/Cabin1	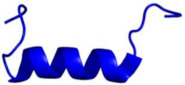 PDB ID: 1N6J; CABIN1 in complex with myocyte-specific enhancer factor 2B (MEF2B) (MEF2B removed for clarity)	PKC hyperphosphorylation of cain/Cabin1 → Inhibition of CaN	Experimental	[56,57]
Guanylyl Cyclase A	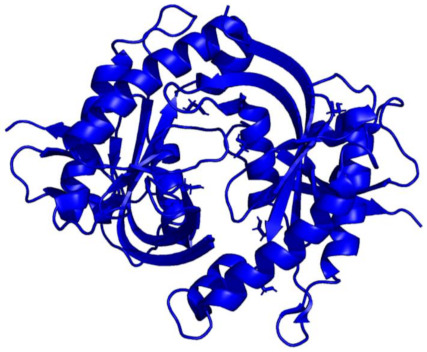 PDB ID: 4NI2	Posited that GCA/cGMP/PKG signaling inhibits calcineurin	Experimental	[58]
Calmodulin-dependent Kinase II (CAMKII)	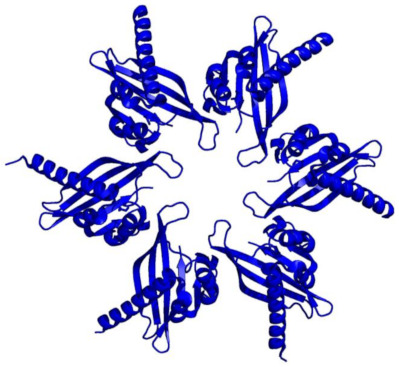 PDB ID: 2UX0	Posited that rise in [Ca^2+^]_i_ → CaMKII activation → phosphorylation/inhibition of CaN	Experimental	[59]
Calcipressin 1 (RCAN1, MCIP1)	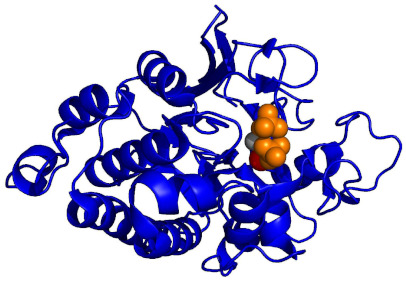 PDB ID: 6UUQ; Orange = PO_4_, Red = Fe, Grey = Zn	Phosphorylation of RCAN1 → binding/inhibition of CaN	Experimental	[60]
Muscle-specific RING finger protein 1 (MuRF1)	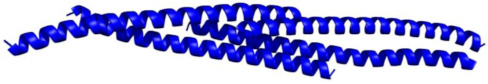 PDB ID: 4M3L	MuRF1 ubiquitinates CaN → CaN degradation	Experimental	[61]

Leads to (→); the Research Collaboratory for Structural Bioinformatic website (rcsb.org) last accessed on 10/22/2021; Protein data bank identification (PDB ID).

**Table 2:** An overview of the roles calcineurin plays in cellular physiology.

Ref.	Cellular Function	System	Results	Conclusion	Conditional Effect
[80]	Adaptive response	Jurkat cells (Immortalized T-cells)	NFATc translocates → nucleus and + newly synthesized NFAT_N_; CsA and FK506 block translocation of NFATc without altering synthesis of NFAT_N_	CsA and FK506 (CaN inhibitors) block NFAT translocation	No stimulants required
[90]	Adaptive response	Jurkat cell from rat kidney	Synergistic activation of α1-α2 adrenergic receptors enhance Na^+^/K^+^ ATPase activity; CaN inhibitors abolish this ATPase enhancement	α-adrenergic stimulation → CaN dephosphorylates Na^+^/K^+^ ATPase → enhanced Na^+^/K^+^ ATPase activity	No stimulants required
[91]	Apoptosis	BHK-21 (lymphocyte not processed by thymus; responsible for antibody production)	CaN transfected cells + 4 h serum deprivation + calcium ionophore = 60% apoptosis; effects obvious in 15–30 min	CaN plays an important role in mediating upstream events in calcium-activated cell death	+ Ca^2+^ and growth factors
[92]	Adaptive response	Jurkat cell	Indirect evidence suggests: CaN + PMA (PKC activator) = inactive IκB via unknown kinase	CaN activation → inactivated IκB = activate NF-κB	No stimulants required
[84]	Adaptive response	B lymphocytes	>5 min @ 200–400 nM [Ca^2+^]_i_ = activation NFATc alone >2 min @ 1 µM [Ca^2+^]_i_ = IκB degradation (inhibitor of NFκB); or JNK1 + PO_4_	Amplitude and duration of [Ca^2+^]_i_ = activation of specific proinflammatory transcriptional regulator (NFAT, NF-κB, or c-Jun N-terminal kinase)	No stimulants required
[93]	Adaptive response	Jurkat cells and dominant negative NFAT transgenic mouse model	Dominant negative NFAT in cultured T-cell = ↓ expression of IL-2 protein Overexpression of CaN = restored IL-2 expression	NFAT = critical component of signaling pathway that regulates IL-2 expression	No stimulants required
[94]	Adaptive response	Rat ventricular cardiomyocytes	+ Ca^2+^ = RyR2-CaN coimmunoprecipitation + 20 mM EGTA = no interaction CaN inhibitors inhibit RyR2 Ca^2+^ release in CM	CaN-RyR2 interaction may modulate calcium release in rat hearts	No stimulants required
[95]	Differentiation	Skeletal muscle cells (C2C12 and Sol8)	+ CaN or + NFATc3 ↑ MyoD (myoblast determination protein) → myogenesis (formation of muscle) CaN inhibitor (cain) blocks differentiation	CaN signaling contributes to initial myogenic myocyte differentiation through NFATc3-dependent mechanism	No stimulants required
[96]	Apoptosis	Cultured CM from 1-day Wistar rats and dominant negative CaN transgenic mice	Isoproterenol induces apoptosis Ca_V_1.2 blocker (1 μM nifedipine) inhibit apoptosis CsA and FK506 inhibit apoptosis	CaN and LTCC play a critical role in iso-induced apoptosis of rat CM	Isoproterenol
[85]	Neonatal rat CM primary cultures 10T12/2 fibroblasts and COS-7 cells	Harlan Sprague–Dawley	Constitutively active CaN = ↑MAKP1 expression and p38-MAPK inactivation in CM	CaN indirectly inactivates p38 MAPK CaN + MEK1, MKK6, or MKK7 = ↑MKP-1 protein levels	Phenylephrine
[97]	Differentiation	Chondrogenic cells (RCJ3.1C5.18)	Ca^2+^ ionophore (ionomycin) induces limb buds in mouse embryos CsA inhibits cartilage development (both +/− ionomycin) + ionomycin → dephosphorylated NFAT4 = activated NFAT4	CaN/NFAT4 activates bone morphogenetic protein expression → chondrogenesis (cartilage formation)	Ionomycin
[86]	Apoptosis	Neonatal rat cardiomyocytes with Adenovirus modification	CaN dephosphorylates ASK1 = promotes dissociation from 14-3-3 protein → activates ASK1 CaN and ASK1 cooperatively regulate CM apoptosis	CaN and ASK1 contribute to a feedback regulatory mechanism involved with signaling CM apoptosis	No stimulants required
[98]	Apoptosis	CaNβ double knockout mouse model	Removal of CaNβ (β isoform of CaN) accelerated spontaneous T-cell apoptosis IL-7 and/or IL-15 treatment inhibited spontaneous apoptosis of T cells Constitutively active CaN stimulated Bcl-2 reporter activity	CaN enhances B-cell lymphoma (Bcl-2) expression overexpression of CaNβ → restored Bcl-2 protein expression = enhanced T cell survival	No stimulants required
[99]	Growth	Neonatal rat ventricular myocytes PS120	Na^+^/H^+^ Exchanger 1 (NHE1) interacts with CaN NHE1 → activates CaN → NFAT translocates nucleus	NHE1 activity → local elevated pH → enhanced CaN activity → NFAT signaling → cardiomyocyte enlargement (hypertrophy)	Overexpression of NHE1
[100]	Adaptive response	Bovine pulmonary atrial endothelial cells Human pulmonary atrial endothelial cells HEK-tsA201	CaN dephosphorylates MYPT1 MYPT1 interacts with CaN (pulldown) CsA + thrombin → sustains MYPT1 and myosin phosphorylation → reduced endothelial electrical resistance	CaN dephosphorylation of cofilin and Myosin Phosphatase → may improve endothelial barrier function (i.e., increased electrical resistance)	Thrombin

leads to (→); decrease (↓); and increase (↑).

**Table 3:** Roles of calcineurin in cardiac disease.

Ref.	System	Animal Model	Conditional Requirement	Results	Conclusion
[109]	Rat	Tg expression of constitutively active CaN Tg expression of constitutively active NFAT3 (nuclear)	Angiotensin II or Phenylephrine	NFAT3 interacts with GATA4 (transcription factor) Combination of CaN + GATA4 + NFAT3 activates (~150×) brain natriuretic peptide promoter CsA and FK506 inhibit induced hypertrophy (angiotensin II and phenylephrine) Constitutively active CaN or NFAT → heart dilation and hypertrophy	Constitutively active CaN → upregulates NFAT3 → cardiac dilation and hypertrophy
[110]	Rat	Neonatal Wistar–Kyoto	Angiotensin II, phenylephrine, or 1% fetal bovine serum	CaN (β-isoform) activity increased by hypertrophic stimuli ↑expression of Cain or AKAP79 inhibitory domain reduce stimulated enlargement of CM surface area CsA and FK506 reduce stimulated enlargement of CM surface area	Increased CaN activity increases CM surface area Decreased CaN activity can prevent an increase in CM surface area
[111]	Mouse	Sprague–Dawley mouse model of load-induced hypertrophy	Abdominal aortic banding (AAB)	↑CaN activity, ↑coimmunoprecipitation of calmodulin-CaN complex CsA prevented AAB-induced gain in heart to body weight ratios	In pressure overload hypertrophy, CaN-CaM interaction is enhanced CsA can attenuate induction of hypertrophy
[112]	Rat and mouse	Sprague–Dawley rat neonate mouse model of ischemia/reperfusion mouse model of constitutively active CaN	DNA damage inducing agent (staurosporine or 2-deoxyglucose)	Constitutively active CaN = ↑CM surface area + protects from induced DNA damage CaN (α-isoform) reduces DNA damage by multiple mechanisms Both NFAT3 and protein kinase B can contribute to CaN-driven protection from DNA damage	CaN may protect CM from apoptosis in vitro and in vivo CaN inhibition is not sufficient to induce apoptosis in vivo
[113]	Mouse	Tg expression of Cain/Cabin-1 Tg expression of AKAP79 inhibitory domain	Isoproterenol	Genetic inhibition of CaN activity in the heart reduces load-induced hypertrophy	CaN is an important contributor to pressure-overload hypertrophy
[114]	Mouse	Tg expression of: constitutively active CaN, CAIN, or AKAP79 inhibitory domain	No stimulant required	↑CaN activity → cardiac hypertrophy → quickly progresses to heart failure ↑CaN activity → altered LTCC conduction Altered LTCC due to changes Ca^2+^ signaling NOT changes in LTCC gating	CaN induced hypertrophy is associated with enhanced LTCC activity
[115]	Mouse	C57BL/6 Tg removal of NFATc3 Tg removal of NFATc4	Abdominal aortic banding or angiotensin II	NFATc3 deletion reduces pathological CaN-induced hypertrophy NFATc4 deletion does not reduce pathological CaN-induced hypertrophy	Mice lacking NFATc3 are partially protected from cardiac hypertrophy in response to CaN activation
[116]	Mouse	Tg overexpression of K_V_4.2 N-terminus	K_V_4.2 N-terminus overexpression	Reduced I_to_ leads to ↑CaN activity, ↑AP duration, and ↓SERCA2a expression Overexpression of K_V_4.2N-terminus leads to ↑heart to body weight ratio CsA or verapamil removed this effect and restored SERCA2a expression	In mice, reduced I_to_ leads to enhanced Ca^2+^ cycling and hypercontractility
[117]	Mouse ventricle	Tg expression of: constitutively active CaN or constitutively active NFAT3 (nuclear)	Overexpression of chronically active CaN and isoproterenol	↑CaN activity or ↑NFAT3 = ↑I_Ca_ current density, ↑I_Ca_ inactivation kinetics, ↑heart contractility, ↓K_V_2.1 expression, ↑AP duration, ↓heart rate, and ↓β-adrenergic stimulation	CaN overexpression causes hyperdynamic cardiac remodeling
[118]	Rat ventricle	Sprague–Dawley	Phenylephrine or Verapamil and PKG overexpression	↑Phenylephrine leads to ↑LTCC activity leads to ↑NFATc3 translocation leads to ↑transcriptional activity ↑Nitric oxide → activation of PKG I → blunts phenylephrine effect Nitric oxide/cGMP diminishes CaN-induced CM size increase	Nitric oxide/cGMP activation of PKG I inhibits hypertrophic CaN-NFAT pathway in CM
[119]	Mouse	Tg expression of dominant negative JNK1/2 (C57BL/6 background, FVB/N strain)	TAC	↓JNK1/2 (kinase) activity = ↑heart to body weight ratio, ↑induced hypertrophy, ↑CaN-NFAT signaling	Dominant negative JNK (kinase) enhances CaN-NFAT signaling → cardiac hypertrophy
[120]	Mouse	Tg expression of: dominant negative p38α, dominant negative MKK3, or dominant negative MKK6	Aortic banding, angiotensin II, isoproterenol infusion, or phenylephrine infusion	dnp38α, dnMKK3, or dnMKK6 = ↑heart to body weight ratio, ↑heart dilation, ↑cross sectional area Angiotensin II, phenylephrine, or isoproterenol effects are enhanced in dominant negative animal models with aortic banding ↓p38α activity + CaN A transient transfection = ↑NFAT translocation to nucleus	dnp38α, dnMKK3, or dnMKK6 → enhance CaN-NFAT translocation and transcription (luciferase activity)
[58]	Mouse	Tg knockout of guanylyl cyclase A (C57BL/6 and 129SVj)	No stimulants required	K.O. guanylyl cyclase A = ↑CaN activity, ↑NFATc3 translocation, ↑GATA4 DNA binding, ↑ANP and BNP (mRNA), ↑collagen I and III, ↑Fibronectin K.O. guanylyl cyclase A + FK506 attenuates observed modifications	Disruption of guanylyl cyclase A activates cardiac CaN-NFAT pathway FK506 attenuates fibrosis and hypertrophy
[121]	Rat	Overexpression of constitutively active CaN Sprague–Dawley	Overexpression of CaN	↑CaN activity = ↑cell capacitance, ↑^3^H-leucine uptake, ↑mRNA encoding KV4.2, ↑KV4.2 current, and ↑chord conductance (each ion’s contribution to membrane potential) cain = removed all enhancement effects	Constitutively active CaN increases cardiac K_V_4.2 current
[122]	Mouse	Tg overexpression of ZAKI-4β (endogenous CaN inhibitor)	TAC	TAC + overexpression of ZAKI-4β (CaN inhibitor) = ↓PLB-PO_4_, ↓SERCA2a protein, ↓NCX protein, ↓RyR2-PO_4_, and ↑Left ventricular end diastolic pressure → increased myocardial stiffness	In TAC mouse model, CaN inhibition = ↓hypertrophy but does not prevent diastolic dysfunction
[59]	Rat and feline	Tg expression of: constitutively active CaMKII or dominant negative CaMKII	No stimulants required	↑CaMKII activity = ↓NFATc3 accumulation in nucleus, ↓fractional shortening, ↑DNA damage ↑Ca^2+^ leads to ↑NFAT accumulation in nucleus ↑CaMKII activity leads to ↑NFAT phosphorylation CaN inhibitors remove these effects	↑CaMKII activity → phosphorylation of CaN → reduced NFAT accumulation in nucleus
[82]	Mouse	Tg K.O. of CaN B subunit (C57BL/6 background)	No stimulants required	Deletion of CaN B-subunit = fatal < 1 day after birth, ↓NFAT activity, ↓mRNA of ion-handling genes, ↓capillaries per myocyte, ↓active force generation, ↑CM size	CaN signaling is linked to control of cardiac contractility, rhythm, and expression of Ca^2+^ handling proteins

Leads to (→); decrease (↓); increase (↑); transgenic (Tg); Transaortic Constriction (TAC); Phosphate (PO_4_); Sarco/endoplasmic reticulum calcium ATPase (SERCA); knock out (K.O.).

**Table 4:** List of CaN substrates found in a cardiomyocyte.

Ref.	Substrate	K_D_	Molecular Effect	Cellular Effect	Physiological Effect(s)
[56,109,111,138,139,140]	Nuclear factor of activated T cells 1-4 (NFAT1-4)	N/A	Dephosphorylation of NFAT_c_	Relocalization of calmodulin-CaN-NFAT complex to nucleus → bind to NFAT_N_ → expression of interleukin (cytokine)	Initiation of inflammatory immune response and/or cardiac hypertrophy
[141,142]	ATP sensitive K^+^ channel	N/A	↑ Ca^2+^ = ↓K_ir_6.1 current Constitutively active CaN Aα= ↓K_ir_6.1 current	N/A	N/A
[100]	Myosin phosphatase (MYPT1)	94 nM	CaN dephosphorylates ^32^P-MYPT1 MYPT1-CaN interaction supported by pulldown, colocalization, and SPR experiments	N/A	N/A
[96,102]	L-Type Ca^2+^ Channel	N/A	Inside-out patch + CaN inhibits LTCC conduction	N/A	N/A
[90]	Na^+^/K^+^ ATPase	N/A	+FK506 or CaN peptide inhibitor = no effect of Oxymetazoline (Na^+^/K^+^ ATPase activator)	N/A	N/A
[92]	IκB	N/A	Constitutively active CaN + ionomycin = ↑NF-κB reporter activity	N/A	N/A
[94]	RyR2	N/A	Ca^2+^-dependent CaN binding to RyR2 → inactivation of RyR2	N/A	N/A
[86,87]	ASK1	N/A	CaN B dephosphorylates ASK1 (S967) → ASK1 dissociation from 14-3-3 protein → ASK1 activation	Apoptosis	N/A
[143]	Myopodin	N/A	CaN dephosphorylates myopodin → hindered myopodin binding to 14-3-3β Prevention of myopodin binding to importin α	N/A	N/A
[144,145]	Calsarcin1	N/A	Calsarcin 1 binds to CaN A → formation of trimer with α-actinin	Localization of CaN to z-line of cardiomyocyte	Inhibition of calcineurin in hypertrophic signaling

Not available (N/A); leads to (→); decrease (↓); increase (↑).
